# A Model to Explain Plant Growth Promotion Traits: A Multivariate Analysis of 2,211 Bacterial Isolates

**DOI:** 10.1371/journal.pone.0116020

**Published:** 2014-12-26

**Authors:** Pedro Beschoren da Costa, Camille E. Granada, Adriana Ambrosini, Fernanda Moreira, Rocheli de Souza, João Frederico M. dos Passos, Letícia Arruda, Luciane M. P. Passaglia

**Affiliations:** 1 Departamento de Genética, Instituto de Biociências, Universidade Federal do Rio Grande do Sul, Porto Alegre, RS, Brazil; 2 UNIVATES, Lajeado, RS, Brazil; 3 Empresa de Pesquisa e Extensão Agropecuária de Santa Catarina (EPAGRI), Lages, SC, Brazil; University of Milan, Italy

## Abstract

Plant growth-promoting bacteria can greatly assist sustainable farming by improving plant health and biomass while reducing fertilizer use. The plant-microorganism-environment interaction is an open and complex system, and despite the active research in the area, patterns in root ecology are elusive. Here, we simultaneously analyzed the plant growth-promoting bacteria datasets from seven independent studies that shared a methodology for bioprospection and phenotype screening. The soil richness of the isolate's origin was classified by a Principal Component Analysis. A Categorical Principal Component Analysis was used to classify the soil richness according to isolate's indolic compound production, siderophores production and phosphate solubilization abilities, and bacterial genera composition. Multiple patterns and relationships were found and verified with nonparametric hypothesis testing. Including niche colonization in the analysis, we proposed a model to explain the expression of bacterial plant growth-promoting traits according to the soil nutritional status. Our model shows that plants favor interaction with growth hormone producers under rich nutrient conditions but favor nutrient solubilizers under poor conditions. We also performed several comparisons among the different genera, highlighting interesting ecological interactions and limitations. Our model could be used to direct plant growth-promoting bacteria bioprospection and metagenomic sampling.

## Introduction

Plant growth-promoting bacteria (PGPB) are microorganisms that are naturally found inside and around plant roots. These microorganisms participate in complex ecological interactions in the rhizosphere, where they can influence the health, growth and stress response of their host plants [1]. PGPB can be used as inoculants for crop plants aiming at sustainable food production. In some cases, the use of these bacteria can reduce chemical fertilizer usage up to 50% [2], which represents a huge benefit to the environment because chemical fertilizers are polluting agents based on finite resources [3]. In fact, research on PGPB has been increasing for years [4], and the use of these bacteria might be the future of modern agriculture [5], either for a biotechnologically intensive or a natural and organic-based approach.

While there are many reports of the successful prospection and use of PGPB, most of the actual interactions that occur in the rhizosphere are unknown, as the soil-plant-microorganism interface is a very complex open system [6]. Because there are many factors affecting rhizosphere dynamics, multivariate statistics for microbial ecology have become a very important tool for understanding the general outcomes that elude univariate statistics and linear relationships [7]. Unfortunately, these methods are not widely used by microbiologists, and even classical statistical tests are absent from several reports. Many papers on PGPB – which are expensive and difficult to obtain – are underexploited. In addition, new molecular biology technologies, such as deep sequencing and microarrays, generate large datasets, requiring advanced statistical analysis [8].

Having at our disposal data from seven different studies that shared a common methodology for bioprospection, we created a databank of 2,211 putative diazotrophic PGPB that were isolated from different crops. We discovered interesting patterns in the soil-plant-microorganism interface that were not clear in the independent studies upon which this paper was based. We propose a model that suggests that plants permit an endophytic relationship with associated bacteria based on the plant nutritional needs and on the bacterial plant growth-promoting abilities. According to this model, nutrient-solubilizing bacteria are favored under nutrient-poor conditions, while hormone-producing bacteria are favored under nutrient-rich conditions. These findings can be used to direct the bioprospection of PGPB, genes or metagenomes, and the methodology that is used in these analyses can be replicated by microbiology researchers who have access to a large collection of isolates.

## Materials and Methods

### Dataset compilation

To create the dataset that was used in this work, bacterial collections from six published papers and one personal communication from our group were pooled. These works, although performed independently from each other, shared similar methodology, focusing on the isolation and characterization of PGPB for biotechnological applications. Bacterial isolates were obtained from the rhizospheric soils or roots of rice collected in Cachoeirinha (29°56′51.9′′S, 51°06′46.3′′W) for reference [9]; Aceguá (31°45′11′′S, 54°3′22′′W), Arroio Grande (32°14′19′′S, 53°5′27′′W), Cachoeirinha (29°56′51.9′′S, 51°06′46.3′′W), Santa Vitória do Palmar (33°31′08′′S, 53°22′04′′W), Uruguaiana (29°45′18′′S, 57°05′16′′W), and Viamão (30°04′51′′S, 51°01′22′′W) for reference [10]; wheat collected in: São Borja (28°39′39′′S, 56°00′14′′W), Júlio de Castilhos (29°13′37′′S, 53°40′54′′W), Vacaria (28°30′43′′S, 50°56′02′W), Campina das Missões (27°59′20′′S, 54°50′22′′W), Guarani das Missões (28°08′27′′S, 54°33′29′′W), and Boa Vista do Cadeado (28°35′06′′S, 53°47′57′′W) (Moreira, personal communication); maize collected in: Júlio de Castilhos (29°13′37′′S, 53°40′54′′W), Porto Alegre (30°1′40′′S, 51°13′43′′W), Rio Grande (32°04′54′′S, 52°09′48′′W), Vacaria (28°30′43′′S, 50°56′02′W) and Veranópolis (28°54′3′′S, 51°33′10′′W) for reference [11]; sunflower collected in: Encruzilhada do Sul (30°32′38′′S, 52°31′19′′W)], São Borja (28°39′39′′S, 56°00′14′′W), São Gabriel (30°20′0′′S, 54°19′12′′W)], Vacaria (28°30′43′′S, 50°56′02′′W)], and Viamão (30°04′51′′S, 51°01′22′′W) for reference [12]; apple trees collected in São Joaquim (28°17′36′′S, 49°56′1′′W) for reference [13]; and *Lupinus albescens* grown in arenized and non-arenized areas located between the latitudes of 29°00′S to 31°00′S and longitudes of 54°30′W to 58°45′W for reference [14]. No specific permissions were required for all of these locations and the field studies did not involve endangered or protected species. The analyzed soil chemical characteristics were the pH, clay, organic matter, phosphorous (P) and potassium (K) contents [15]. The characteristics that were considered for the isolates were niche colonization (rhizospheric or endophytic), the amount of indolic compounds (ICs) produced, the halo sizes of bacterial colonies in plate assays for tricalcium phosphate (TCP) the solubilization and siderophores production abilities, the bacterial genera, and the sample origin of the isolate. Nitrogen fixation potential was not quantified for the majoritiy of the isolates, so this PGP trait could not partake in our model. The isolation was performed according to Döbereiner [16]. Isolated diazotrophs are considered putative as some bacteria might survive selective isolation by using cellular N reserves, or scavenging very low N content from the original soil solution. The full dataset for this work is presented in S1 and S2 Tables.

In all of the analyzed studies, rhizospheric isolates were obtained from the soil that was immediately attached to plant roots, and putative endophytic isolates were obtained from surface-sterilized plant roots. Root sterilization was performed in 70% ethanol for 2 minutes and sodium 4.0% hypochlorite for 2 minutes, followed by several water washings. While our surface sterilization procedure might allow the survival of bacteria protected in root crevices or by biofilm, such bacteria nevertheless would have a more intimate colonization of the plant compared to the rhizospheric bacteria. Furthermore, these occasional survivors should not outnumber endophythic bacteria to the point of compromising the results. The halo size of the bacterial colonies in plate assays for TCP solubilization [17] and siderophores production [18] was classified as 1 =  no halo, 2 =  small or average halo size (ranging from 0.1 to 0.6 mm), and 3 =  large halo size (larger than 0.6 mm). The halo size of positive siderophores producers was not registered by one of the authors [11]; therefore, we could only consider the halo size of the non-producing isolates from this dataset in our analysis. Thus, 99 positive siderophores isolates were not analyzed and were considered as missing data regarding their siderophores production ability. Indolic compounds production was determined after 72 h of incubation in King B medium that was supplemented with tryptophan using the Salkowski reagent [19]. The values were reported as micrograms of ICs per milliliter (µg of ICs ml^−1^). The isolates were identified at the genus level by PCR-RFLP and the partial sequencing of the 16 S rRNA gene using the procedures described by Ambrosini *et al*. [12]. In this study, the bacterial genus was considered only if it contained at least 5 isolates. The genera that contained 4 or less isolates or isolates that were not identified at the genus level were pooled as the “rare” portion of the microbiota. This rare portion was composed of 134 unidentified isolates and 57 isolates belonging to 40 genera, as shown in S1 Table.

### Statistical analysis

To classify the different soils samples into poor, average or rich categories, the soil chemical characteristics (pH, organic matter, clay, K and P contents) were analyzed by a Principal Component Analysis (PCA). Afterwards, we tested the PCA soil classification with ANOVA (log-transformed pH, organic matter, clay, and K contents) and Kruskal-Wallis (P contents). The multivariate analysis of the bacterial isolate characteristics was performed by a Categorical Principal Component Analysis (CatPCA).

To associate the categorical data (halo sizes for siderophores production and TCP solubilization abilities, soil richness, and genera), we used the chi-square statistic obtaining the exact p value. When necessary, a Monte Carlo simulation was used to estimate a p value window (the upper and lower borders were always 0.001>p>0.0001). An adjusted standardized residual analysis was used to detect significant individual associations that were reported on a heat map. Comparisons of the ICs production levels according to the soil condition, TCP solubilization and siderophores production were performed with the Kruskal-Wallis nonparametric test followed by Dunn's multiple comparisons, which considers different sample sizes [20]. The comparison of ICs production according to the colonization niche was performed with the Mann-Whitney pair wise comparison. In these analyses, non-ICs producers were not included. As the variance was too high to return meaningful results when comparing the ICs production across genera, we categorized ICs production as low (0–10), average (11–80) and high (80 or more) µg of ICs ml^−1^ and analyzed it as in phosphate solubilization and siderophores production. The differences were considered significant at p<0.05, and to correct for global type I error, we determined a False Discovery Rate of 10% [21]. All hypotheses tests (with sample sizes, p values, degrees of freedom, and false discovery rate) are shown on S3 Table. Additional information on the statistical methods is presented as Supplementary Material (S1 Text).

## Results

Our dataset was composed of 2,211 bacterial isolates classified in 80 genera, with 1,061 endophytic and 1,150 rhizospheric isolates. There were 634 TCP solubilizers, 1,358 siderophores producers, and 1,977 IC producers. These isolates were obtained from 40 different soil samples from seven different plants plus two natural grasslands.

### Multivariate plotting and analysis

#### Soil PCA

**Figure 1:**
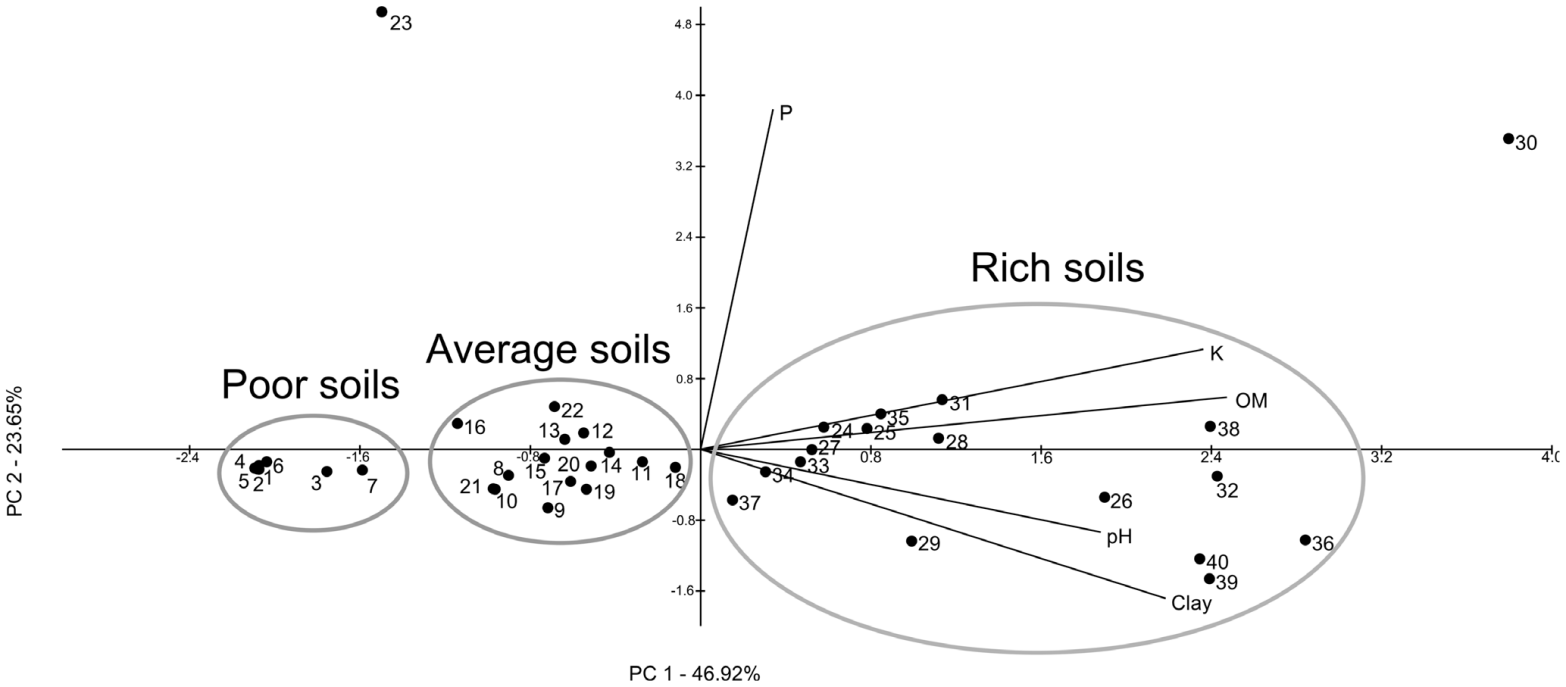
PCA analysis of the soil characteristics from the 40 soils samples (numbered black circles) that were used for bacterial isolation. The percentages show how much variation is explained by each principal component. The soils with higher pH, organic matter (OM), potassium (K), phosphorus (P), and clay (Clay) contents are plotted to the right. There are three clusters along the first principal component (PC1) that grouped the soils by overall richness. Based on these clusters, all 40 of the soil samples were classified according to their overall soil richness: poor, average or rich. The appropriate soil richness was attributed to each bacterial isolate (according to its origin) before further analysis. Supervised statistics of these data on S1 Fig.

The PCA analysis of the soil chemical characteristics allowed us to separate the soil samples into three clusters (Fig. 1). The evaluated characteristics – whose higher values are associated with productive, healthy and rich soils [22] - were plotted on the positive values of the first principal component, with the P contents more associated to the second principal component due to two soils with very high P contents (soils 23 and 30). We considered that these three clusters allowed us to classify the soils from which the bacteria were isolated as poor, average and rich, thereby both grouping and dividing an otherwise very heterogeneous sample origin dataset with mixed plants and farming managements. Soil 23 was considered an average soil, and soil 30 was considered a rich soil. Most of the soils from the poor conditions were from an arenized area that was not used for crop production and that lacks vegetal cover other than *Lupinus* sp., a leguminous plant (Granada *et al.*, 2013). We also performed supervised statistics to test these classifications. For all soil characteristics, richer soils had higher values than poor soils. Average soils presented intermediate values for all soil characteristics, but were statistically similar to rich soils for P contents and pH, and statistically similar to poor soils for clay contents (S1 Fig.).

#### Analysis of the isolates by CatPCA

**Figure 2:**
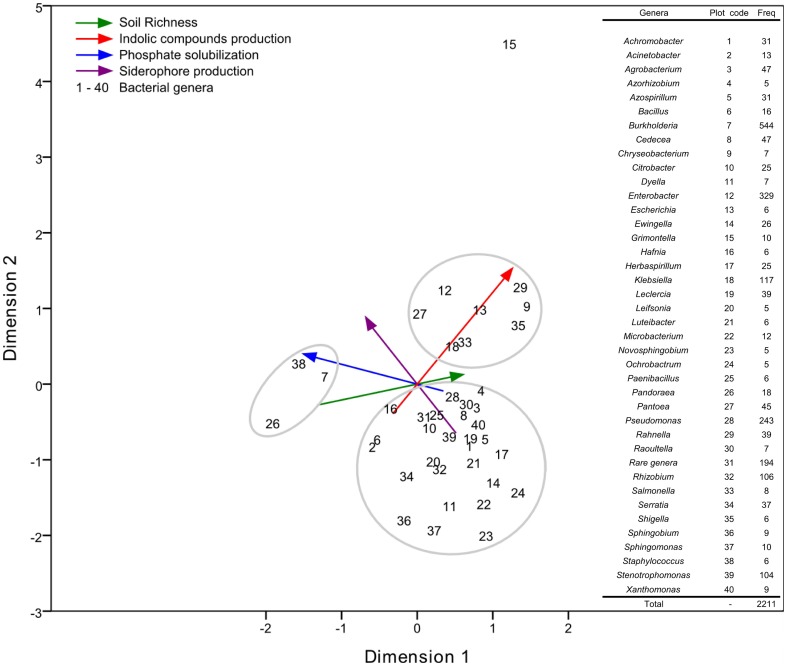
CatPCA analysis of 2,211 bacterial isolates. The indolic compounds production, TCP solubilization, siderophores production and soil richness are shown as colored vectors, with arrows indicating the vector's direction in the plot. The black numbers show the average position of each bacterial genus. In the right column are shown the bacterial genera, the number they represent in the plot (Plot code), and their frequency in the dataset (Freq). Cronbach's alpha value was 0.774.

In the CatPCA analysis (Fig. 2), the soil richness increases towards the positive values of the first dimension (X axis), while the TCP solubilization ability increases with the negative values of the same dimension. Because the vectors (lines) increase in opposite directions, we could say that the best TCP solubilizers would be found in the poorer soils. The ICs production ability of bacterial isolates increases towards the positive values of both dimensions. This result suggests that the ICs production ability of the isolates increases as the soil richness increases and should decrease as the phosphate solubilization ability of the isolates increases. The siderophores production vector is plotted close to the phosphate solubilization vector, suggesting that these vectors could be associated as well. Finally, the different bacterial genera were separated into three different clusters: one associated with high ICs production, another associated with poor soils and high phosphate solubilization, and a larger cluster that does not seem to be associated with a high expression of any of the evaluated PGP traits.

### Hypothesis testing

#### PGP traits and the environment

**Figure 3:**
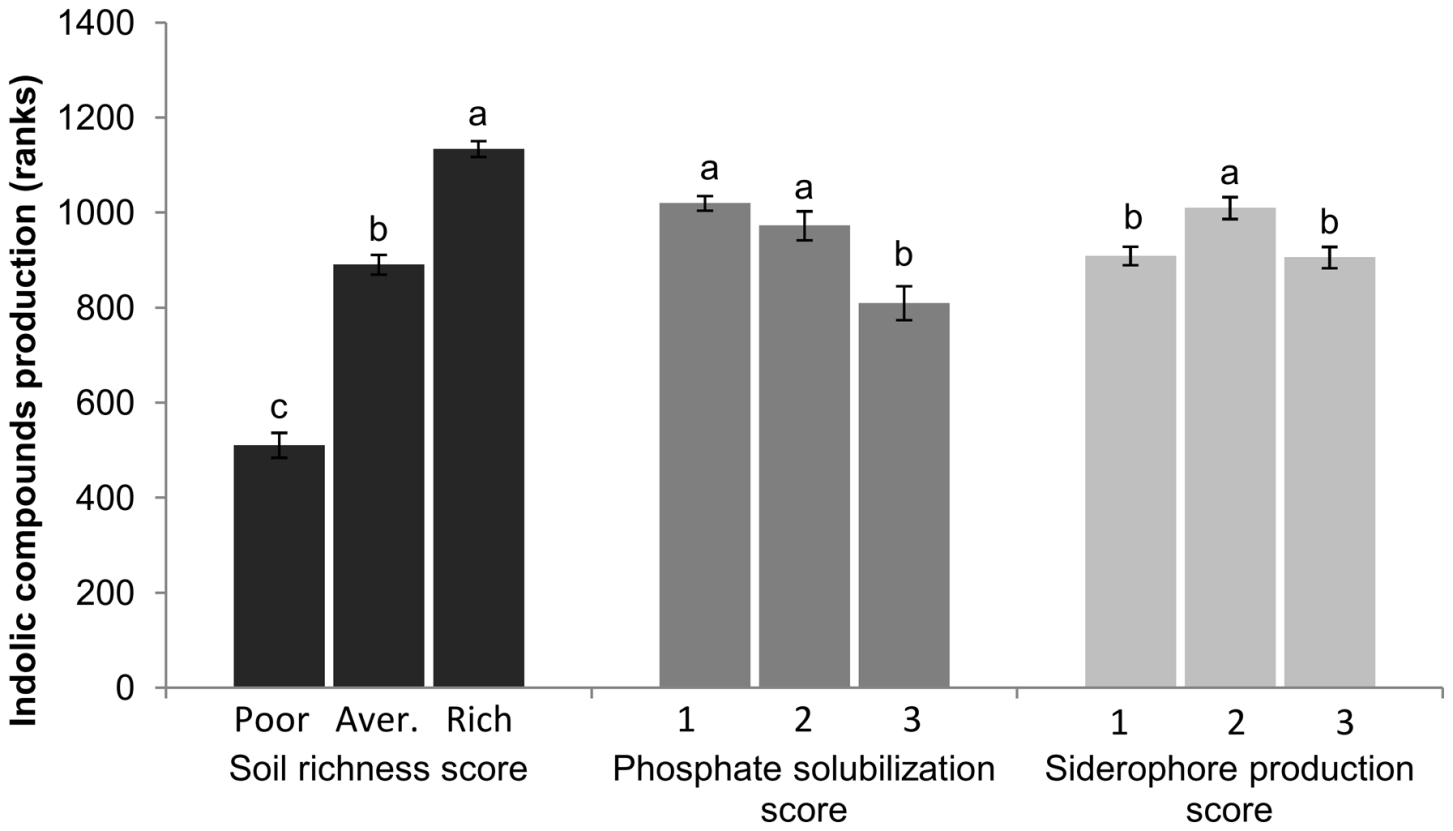
Indolic compound production ability of the isolates (average rank ±1 SE) according to the soil nutrient conditions and TCP solubilization and siderophores production abilities. The phosphate solubilization and siderophores production scores are 1 =  no halo, 2 =  small or average halo, and 3 =  large halo. The soil richness score is according to the PCA analysis (Fig. 1). Different letters show significant differences. Sample sizes and p values are presented on S3 Table.

**Figure 4:**
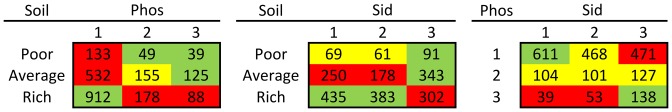
Heat map associations of the TCP solubilization (left) and siderophores production (middle) abilities of bacterial isolates with soil conditions and with each other (right). Phos  =  TCP solubilization, and Sid  =  siderophores production. 1 =  no halo, 2 =  small or average halo, and 3 =  large halo. The red cells  =  less isolates than expected under those conditions, the green cells  =  excessive number of isolates under those conditions, and the yellow cells  =  no significant differences between the observed and expected values. Percentages and residuals are shown in S2 Fig. Sample sizes and p values are presented on S3 Table.

The multiple associations between the PGP traits, bacterial genera and environment were further verified by hypothesis testing. The ICs production ability of the bacterial isolates increases as the soil richness increases (Fig. 3). However, the ICs production ability of the best TCP solubilizers is lower than the ICs production ability of those isolates that did not present a good TCP solubilization capacity. Similarly, the best siderophores producers were not the best ICs producers. The association heat map (Fig. 4 and S2 Fig.) shows that higher TCP solubilization ability of the bacterial isolates was associated with poor soils, and that the richer soils were associated with isolates that presented a lower TCP solubilization ability. Similar associations occurred with siderophores production: the isolates with a strong ability to produce siderophores were associated with poor soils, while those with weak siderophores production abilities were associated with richer soils. Finally, we showed that siderophores production and TCP solubilization abilities have some degree of correlation: there is an excessive number of isolates that were level 1 TCP solubilizers and level 1 siderophores producers, or were level 3 TCP solubilizers and level 3 siderophores producers. At the same time, there was a reduced number of isolates that were level 3 TCP solubilizers and level 1 siderophores producers or that were level 1 TCP solubilizers and level 3 siderophores producers. This observation indicates, for example, that the simultaneous high expression of these two PGP traits in the same bacterium occurs with a greater frequency than expected.

#### Niche effect on the PGP traits

**Figure 5:**
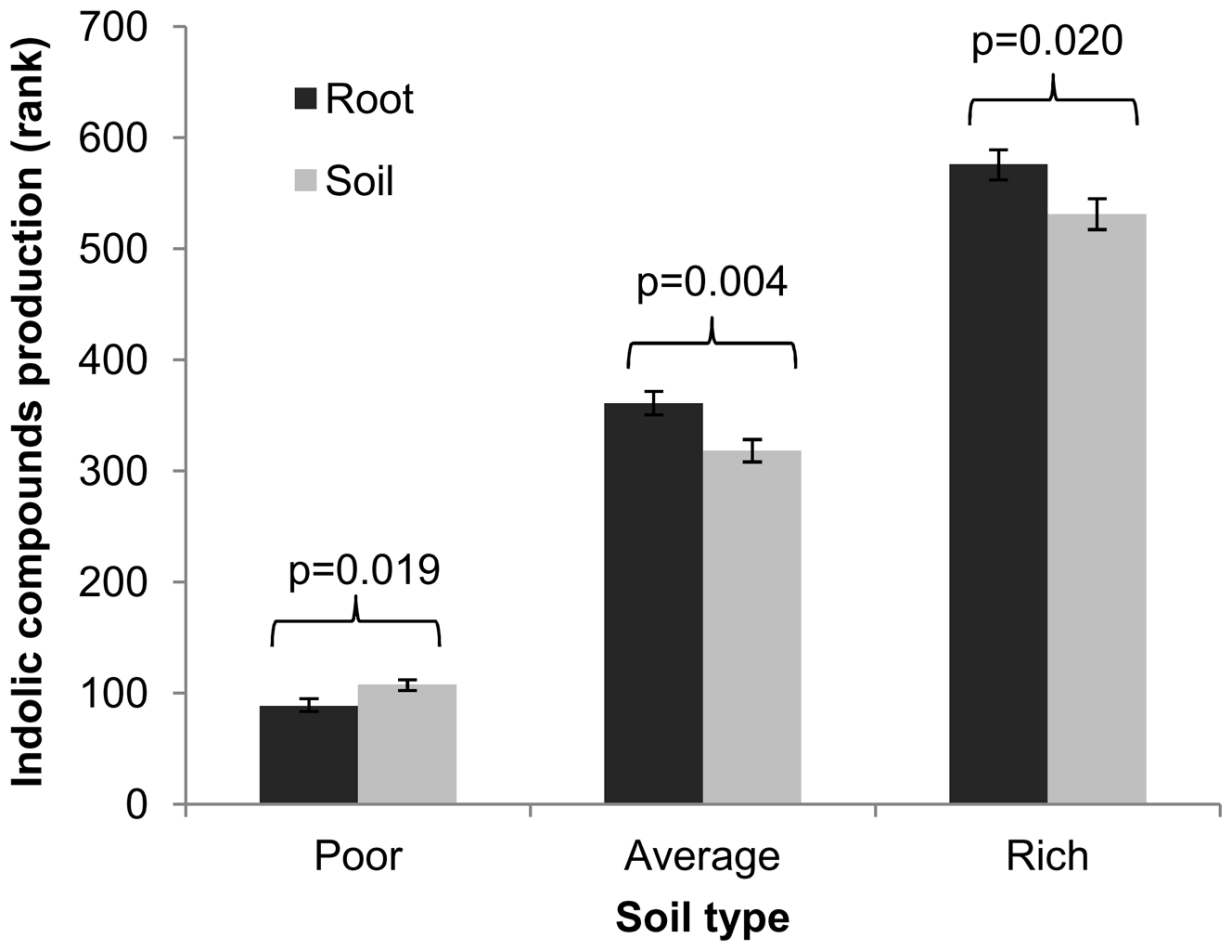
Niche effect on ICs production (average ±1 SE) between endophytic (root) and rhizospheric (soil) isolates under each soil condition. The best ICs producers shift their colonization site according to soil richness. Sample sizes and p values are presented on S3 Table.

**Figure 6:**
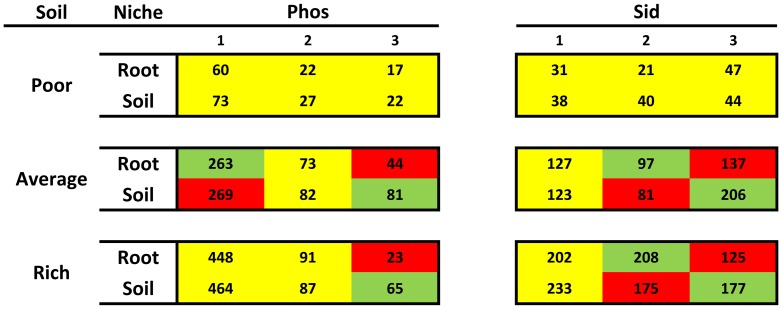
Heat map associations of the TCP solubilization and siderophores production abilities of endophytic (root) and rhizospheric (soil) isolates under each individual soil condition. Phos  =  TCP solubilization, and Sid  =  siderophores production. 1 =  no halo, 2 =  small or average halo, and 3 =  large halo. The red cells  =  less isolates than expected under those conditions, the green cells  =  excessive number of isolates under those conditions, and the yellow cells  =  no significant differences between the observed and expected values. Percentages and residuals are shown in S3 Fig. Sample sizes and p values are presented on S3 Table.

The niche effect – which considers the occurrence of certain bacteria within the plant roots (endophytic) or around the rhizosphere – could not be accurately verified by the CatPCA (see S1 Text). As shown in Fig. 5, the ICs production ability was different between the endophytic and rhizospheric isolates: the best ICs producers were found in the rhizospheric soils of plants that were cultivated in poor soils or were isolated from the roots of plants that were cultivated in average or rich soils. The niche effects on TCP solubilization and siderophores production are shown in a heat map in Fig. 6 and S3 Fig. Apparently, the endophytic and rhizospheric bacterial populations presenting these two PGP traits behaved in a similar manner in poor soils, as these tests were non-significant. However, in average and rich soils, there were more level 3 TCP solubilizers and more level 3 siderophores producers in the rhizospheric soils than there were inside the plant.

#### Bacterial genus association with the PGP traits and the environment

**Figure 7:**
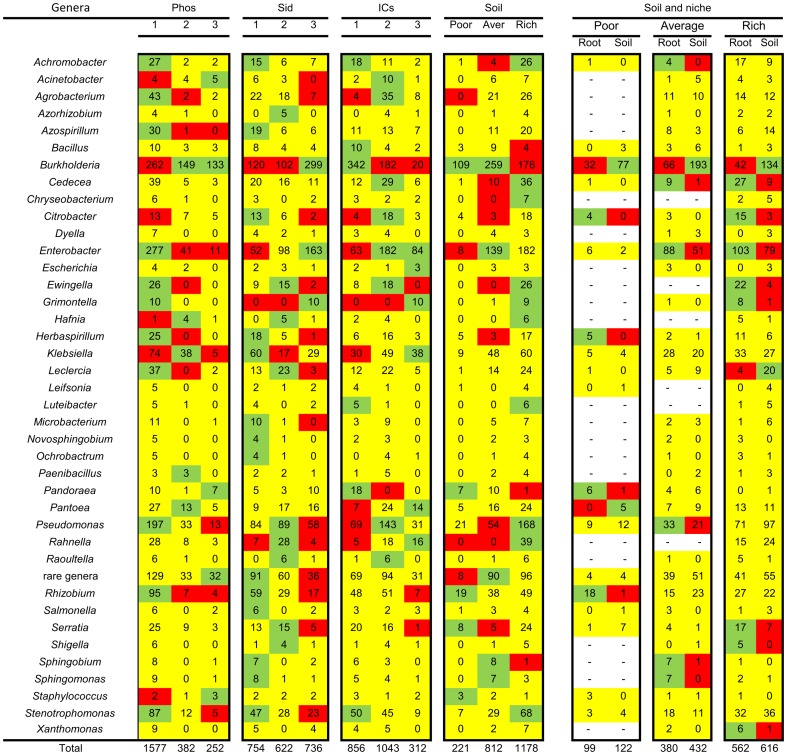
Heat map associations of bacterial genera and PGP traits (left), soil richness (middle), and occurrence of putative endophytic (Root) and rhizospheric (Soil) bacteria under each soil richness condition (right). Phos  =  TCP solubilization, Sid  =  siderophores production, with 1 =  no halo, 2 =  small or average halo, and 3 =  large halo. ICs  =  Indolic compounds production, with 1 =  low (0–10 µg of ICs ml^−1^), 2 =  average (11–80 µg of ICs ml^−1^) and 3 =  high (80 or> µg of ICs ml^−1^). The red cells  =  less isolates than expected under those conditions, the green cells  =  excessive number of isolates under those conditions, and the yellow cells  =  no significant differences between the observed and expected values. “–”  =  an association could not be calculated due to the lack of cases (no expected total marginal values). Percentages and residuals are shown in S4 Fig. Sample sizes and p values are presented on S3 Table.

The bacterial genus association with all of the PGP traits, the environment, and the niche effects can be seen on the heat map in Fig. 7 and S4 Fig. Few bacterial genera (*Burkholderia, Acinetobacter, Hafnia, Pandoreae,* and *Staphylococcus*) presented strains that were associated with a high TCP solubilization ability, while others (*Achromobacter, Agrobacterium, Azospirillum, Enterobacter, Ewingella, Grimontella, Herbaspirillum, Leclercia, Pseudomonas, Rhizobium,* and *Stenotrophomonas*) presented strains that were associated with the non-solubilization ability. Only 29% of the isolates and 77% of the genera presented strains that were able to solubilize TCP. Most of the genera with strains that were associated with high ICs production belonged to the *Enterobactereace* family (*Enterobacter, Escherichia, Grimontella, Klebsiella, Pantoea,* and *Rahnella*), and the most commonly isolated bacterial genus in soil samples, *Burkholderia*, presented strains that were greatly associated with a very low production of ICs. Sixty-one percent (61%) of the isolates and 95% of the genera presented strains that could produce ICs above a residual level (>10 µg of ICs ml^−1^). For siderophores production, few bacterial genera presented strains that were associated with high production (*Burkholderia*, *Enterobacter*, and *Grimontella*), while others (*Klebsiella*, *Stenotrophomonas, Rhizobium, Herbaspirillum*, and *Citrobacter*) presented strains that were associated with a low production of siderophores. Sixty-four percent (64%) of all of the isolates and 100% of all of the bacterial genera presented strains that were able to produce siderophores. Approximately one-third of all of the isolated bacterial genera presented at least one positive association with a PGP trait at a high level. The associations between the genera and soil conditions indicate that many genera were more associated with richer conditions. Only few genera, such as *Burkholderia*, *Pandoreae, Rhizobium, Serratia*, and *Staphylococcus,* were associated with poor soils.

#### Niche effect on the selection of bacterial genera according to the environment

Some bacterial genera might be associated to a colonization niche on some soil richness conditions, but not on others. (Fig. 7 and S4 Fig., right). The strains belonging to the *Burkholderia* genus were found predominantly in the rhizospheric soil samples despite soil richness, whereas the strains belonging to the *Enterobacter* genus were found mostly inside the plant roots (endophytes). The strains belonging to the *Rhizobium, Herbaspirillum*, and *Pandoreae* genera displayed an endophytic behavior only in the samples that were obtained from poor soils. While strains belonging to both the *Rahnella* and *Grimontella* genera were associated with richer soils and presented high levels of ICs production, only those strains belonging to the *Grimontella* genus were more often found inside the plant roots (endophytic). The strains belonging to the *Sphingobium* and *Sphingomonas* genera presented similar PGP traits and behaved endophytically in average soils, which both are associated to. The strains belonging to the *Klebsiella* genus, despite being found very often and presenting a high PGP trait shift (see below), were not associated with any soil condition or colonization niche.

#### Bacterial genera PGP trait shift

**Figure 8:**
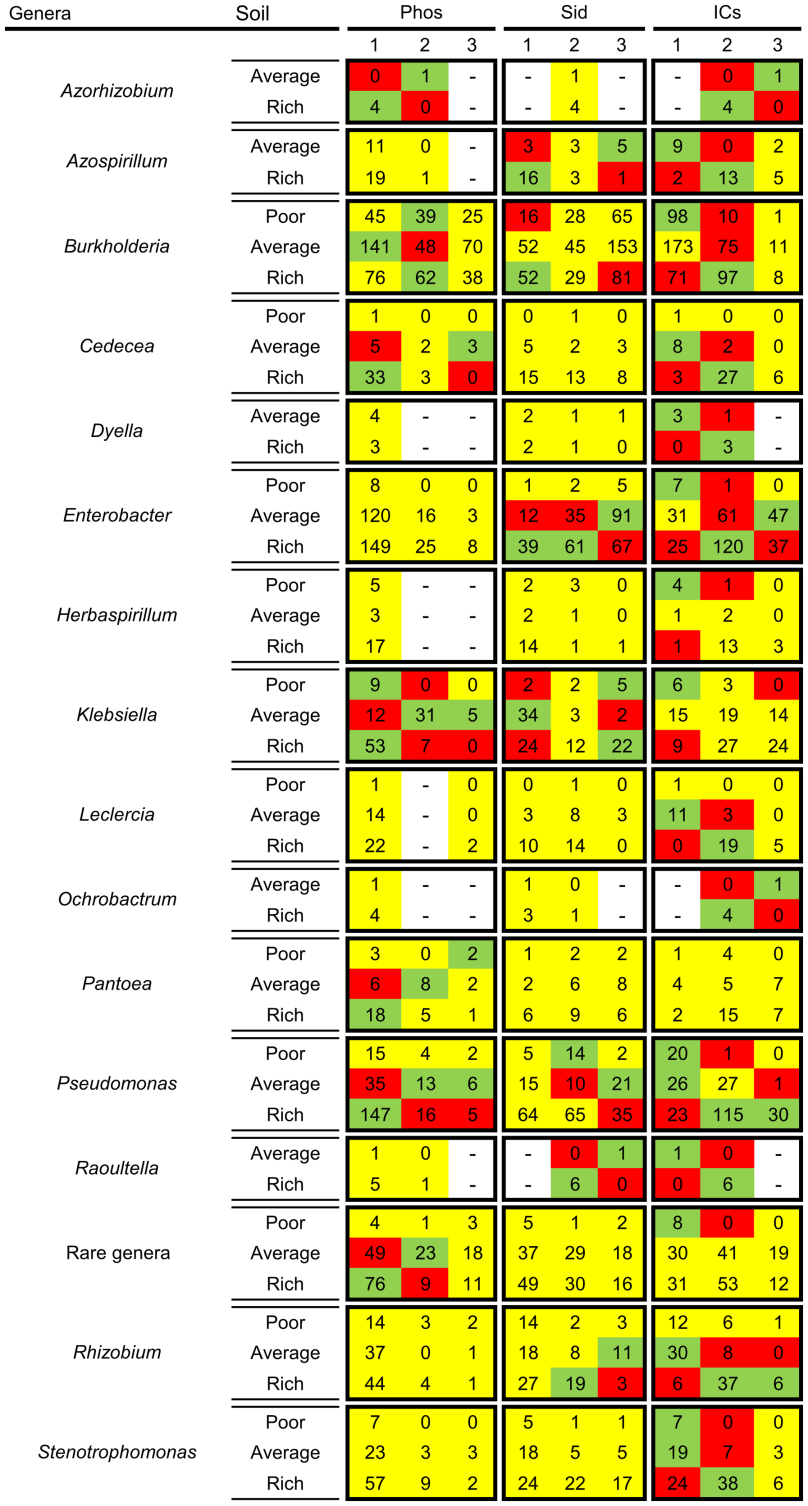
PGP traits of some bacterial strains shifted due to the soil richness. Only those bacterial genera that significantly changed their PGP traits are shown. Each box is a separate chi-square test, with non-significant tests shown entirely in yellow. Phos  =  TCP solubilization, and Sid  =  siderophores production, with 1 =  no halo, 2 =  small or average halo, and 3 =  large halo. ICs  =  Indolic compounds production, with 1 =  low (0–10 µg of ICs ml^−1^), 2 =  average (11–80 µg of ICs ml^−1^) and 3 =  high (80 or> µg of ICs ml^−1^). The red cells  =  less isolates than expected under those conditions, the green cells  =  excessive number of isolates under those conditions, and the yellow cells  =  no significant differences between the observed and expected values. “–”  =  an association could not be calculated due to a lack of cases (no expected total marginal values). Percentages and residuals are shown in S5 Fig. Sample sizes and p values are presented on S3 Table.

The genera presented on Fig. 8 have shifted the occurrence of some PGP abilities according to the soil richness. Fig. 8 and S5 Fig. show independent chi-square tests for each genus that presented a significant deviation from the expected values due to the soil condition on at least one PGP trait. A PGP trait increases under a given soil condition if the number of level 3 producers is larger than expected and/or the number of level 1 producers is lower than expected. Likewise, a PGP trait decreases when the opposite occurs.

For example, the strains belonging to the *Raoultella*, *Azospirillum* and *Rhizobium* genera had an increase in ICs production in rich soils and presented a decrease in siderophores production. In average soils, however, there was a decrease in ICs production and an increase in siderophores production. However, the TCP solubilization ability was unchanged. The strains belonging to the *Pseudomonas* and *Cedecea* genera had an increase in ICs production in rich soils, while they decreased their TCP solubilization ability. For average soils, however, there was a decrease in ICs production and an increase in phosphate solubilization. For strains belonging to the *Burkholderia, Klebsiella, Leclercia*, *Stenotrophomonas*, *Herbaspirillum*, and *Dyella*, genera there was an increase in ICs production in richer soils and a decrease in poor and average soils. Of these isolates, however, only the *Burkholderia* isolates showed a decrease in siderophores production in richer soils, and only *Klebsiella* isolates showed a decrease in TCP solubilization in richer soils. We note that the strains belonging to the *Burkholderia*, *Klebsiella* and *Pseudomonas* genera were the most variable in their PGP abilities in response to the soil conditions, as all of their three PGP traits that were evaluated in this study changed according to the environmental conditions.

**Figure 9:**
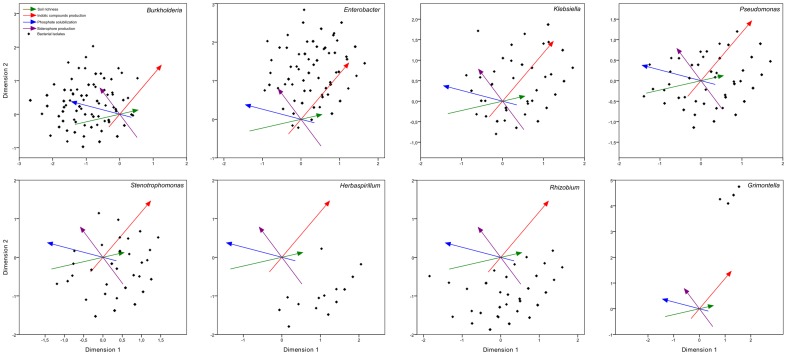
CatPCA analysis of 2,211 bacterial isolates (the legend and interpretation are similar to those of Fig. 2). The genera *Burkholderia*, *Enterobacter*, *Klebsiella*, *Pseudomonas*, *Stenotrophomonas*, *Herbaspirilum*, *Rhizobium*, and *Grimontella* are represented one at a time. Each black dot represents an isolate, but isolates with the same characteristics are stacked on the same dot.

Approximately one-third of the studied bacterial genera presented PGP trait shifting, and in most cases PGP trait shifting follows our model (23 of the 29 cases). Exceptions were found in the siderophores production of strains belonging to *Klebsiella* genus, where the strains with a high production of siderophores were associated with rich soils, and for the ICs production levels of strains belonging to the *Azorhizobium* and *Ochrobactrum* genera, where the strains with lower ICs production levels were associated with richer soils. To better visualize the PGP trait variability of *Burkholderia*, *Enterobacter*, *Klebsiella*, *Pseudomonas*, *Stenotrophomonas*, *Herbaspirilum*, *Rhizobium*, and *Grimontella* genera we created additional CatPCA plots (Fig. 9). In Fig. 9 a single genus is visually displayed, showing all isolates from that genus. All the other 39 genera were visually suppressed, but still take part in the mathematical construction of the plot.

## Discussion

Multivariate methods are very useful in microbial ecology. These methods permit a massive reduction in complexity while simultaneously exploring several research questions. Despite some limitations, such as the use of cultivable bacteria, the halo size and ICs quantification through spectrometric analyses, our study presents the largest databank of bacterial isolates displaying different plant growth-promoting abilities that we are aware of.

### A model to explain the occurrence of PGP traits

The CatPCA analysis demonstrated that the bacterial ICs production levels increase as the soil richness increases. Meanwhile, the bacterial TCP solubilization ability increases as the soil richness decreases, and the bacterial siderophores production seems to be correlated with the TCP solubilization ability (Fig. 2). The interaction between the bacterial ICs production and TCP solubilization abilities with increasing nutrient levels has already been proposed in a previous work [9] but was here confirmed with a sample that was 12-fold larger in size (Fig. 3). While the TCP solubilization ability was expected to be higher or more important in the soils with lower P contents [23]–[26], the association of higher levels of ICs production with richer soils (Fig. 3) was never suggested by other authors. The association of siderophores production with soil richness was also not reported elsewhere.

We detected a decrease in siderophores production by bacterial isolates in richer soils (Fig. 4). Although siderophores-producing bacteria can be often found in Fe-limited soils [26] the iron concentrations in the sampled soils were not measured in this study. Nevertheless, siderophores have functions other than iron homeostasis. Siderophores may bind to more than 16 different metal ions, either for nourishment or to avoid metal toxicity [27], [28], in addition to being able to reach an optimum production in nutrient scarcity conditions [29]. In a diazotrophic *Azotobacter vinelandii* strain, siderophores were produced to capture Mo and V metals for nourishment [30] even in the presence of Fe [31]. As organic matter and clay act as ligands for metals, affecting their availability [32], bacterial siderophores production could be associated with these variables. Acidic sandy soils with low organic matter content, such as those from the poor soil conditions described here, are more susceptible to heavy metal toxicity [33] and could have favored bacterial strains that displayed greater siderophores production for toxicity alleviation. Thus, the increased number of bacterial strains that presented larger halo sizes for siderophores production in poor soils may be related to both general metal acquisitions for nutrients and toxicity alleviation. Furthermore, siderophores action could liberate usable molecules that are attached to binding metals, such as FePO_4_, which potentially acts as a source of P [34]. We found a correlation between the halo sizes of bacterial colonies for siderophores production and TCP solubilization on indicator media (Fig. 4), which might be caused by the Ca binding by siderophores. The occurrence of this correlation in nature is currently being further investigated with an updated phosphate solubilization assay [35]. As 100% of the studied genera presented strains displaying the ability to produce siderophores (Fig. 7), we confirm that this PGP trait is widespread in rhizospheric bacteria [36], similarly to the ability to produce ICs [37], [1]

The inverse correlation between ICs production and TCP solubilization is not deterministic or prohibitive: 23 strains in our database (5 of them belonging to the *Burkholderia* genus) produced more than 80 µg of ICs ml^−1^ and simultaneously showed large halos on TCP medium (S1 Table). Chaiharn and Lumyong [38] isolated 216 bacterial strains where the best ICs producer was also the best phosphate solubilizer, while Bianco and Defez [39] showed that a genetically engineered *Sinorhizobium* strain that overproduced ICs improved its phosphate solubilization ability. While a single strain could enhance plant growth simultaneously via these two mechanisms, our results suggest that the average phosphate solubilization and average ICs production of diazotrophs in soil are under the proposed interaction: the best ICs producers are not the best TCP solubilizers. It seems that the driving mechanism behind this correlation is ecological and not molecular and it is better visualized in a soil richness gradient [9]. Spaepen and Vanderleyden [40] reviewed the molecular aspects of ICs production, and the only reported environmental constrain was that carbon limitation is required for ICs biosynthesis in *Azospirillum brasilense* [41].

Plants have a great effect on the microbial species that surround their roots due to the action of exudates [42]. The rhizosphere is a complex and competitive environment where the bacterial colonization of the interior of the roots is under higher control of the plant and provides more benefits for the bacteria [6], [43]. ICs production by bacteria is also greatly controlled by plants. Not only do plants actively exude tryptophan [37], a necessary amino acid in the tryptophan-dependent indolic acetic acid production pathway, but they might even induce the expression of tryptophan permease genes in bacteria [44]. Rhizospheric bacteria produce more ICs than do bulk soil bacteria [45], but in this study, we expand this effect to endophytic bacteria (Fig. 5) and determine the conditions of its occurrence, corroborating our hypothesis that in richer soils, the best IC producers are endophytic, while the best nutrient solubilizers are not (Fig. 6), due to active plant influence and selection. This suggests that the plant permits interaction with endophytic or rhizospheric bacteria displaying different PGP abilities according to its nutritional status [6], [9]. It is important to notice that plants have limited space and resources and cannot, therefore, select both good ICs producers and good nutrient solubilizers when these groups are composed of different bacteria. This finding addresses a critical research need raised by Gray and Smith [43], as it demonstrates that differences between ePGPB and iPGPB in relation to indolic acetic acid production may be found across a soil richness gradient. Nutrient solubilizers do not necessarily have to live in the rhizosphere to aid nutrient acquisition by plants. Bacteria may act as phosphate solubilizers and metal chelators endophytically [46]-[48]. Thus, endophytic nutrient-solubilizing bacteria from poor soils may act on nutrient acquisition directly, perhaps more actively than rhizospheric bacteria that are closer to the soil nutrients themselves.

We could also identify three clusters of bacterial genera scattered on the CatPCA plot: one small group of genera that were associated with nutrient solubilization, another group associated with phytohormone production, and a larger third group that could be associated with other, non-screened PGP traits (Fig. 2). We suggest that good nutrient solubilizers are more common under limited nutrient conditions in which the plant would benefit most from bacteria that help its nourishment. Additionally, good growth hormone producers are more common under nutrient-rich conditions in which the plants are not starving and may use bacterial secondary metabolites for improved shoot and root growth. This situation is reinforced by the observation that TCP solubilization and growth hormone production are inversely related. The large cluster with other PGPB include nitrogen fixers, such as strains belonging to the *Herbaspirillum* and *Rhizobium* genera (Fig. 9), and should include bacteria with other PGP traits that were not tested in this study (for example, nitrogen fixation, ACC deaminase activity, and disease resistance) as well as soil bacteria that do not act as PGPB. Despite the large number of papers evaluating soil bacteria functional groups [49]–[51] or reviews regarding simultaneous ICs production and nutrient solubilization [1], [4], [52]–[56], the clustering and interactions of hormone producers and nutrient solubilizers was never suggested before.

### Highlights of specific genera that are associated with the PGP traits, niche and environment

Several interesting associations can be found in the heat maps in Figs. 7 and 8. Some of these associations are described below, and can also be noticed on Fig. 9. We believe that our highlights could help direct bioprospection, suggest specific research questions, and illustrate the behavior of some bacterial genera in the plant-soil interface.

Strains from the *Burkholderia* genus are a dominant component of many soil ecosystems [57]. These strains are often found in adverse or unprovided environments, such as in Al-toxic soils [58] or forest to grassland vegetation shift where the soil organic matter content sharply decreases [59]. This genus has strains that were previously characterized as mostly external to the root tissue [43] and very capable of solubilizing nutrients [48], [56], [60], [61]. *Burkholderia* strains present exceptional metabolic and functional diversity [62], possibly provided by their genomes of 4–9 Mb [57]. Here, we demonstrate that *Burkholderia* is a very common genus living mostly outside the root tissue and that is more associated with poor soils and acts as a good nutrient solubilizer, in addition to having a wide versatility and environmental adaptability – all of which agrees with current knowledge. However, Park and Gurian-Sherman [57] stated that the role of siderophores production by *Burkholderia* in root colonization has not been investigated. Here we provide evidence that shows that the siderophores production potential by *Burkholderia* strains decreases as the soil richness increases (Fig. 8). Also, in rich soils, the best siderophores producers were found in the rhizosphere rather than inside plant tissues – a tendency that disappears under poor soil conditions (Fig. 6). Furthermore, when we consider only *Burkholderia* isolates in an analysis that is similar to the one presented in Fig. 6, we could observe that, in poor soils, the best siderophores producers are actually more often found inside the plant than in the rhizosphere (S6 Fig.). We also depict *Burkholderia* strains as poor indolic compound producers that are more often found in the rhizosphere than inside the plant despite soil richness conditions (Fig. 7). This behavior of the *Burkholderia* genus was not previously described [1], [5], [43], [63].

The *Enterobactericeae* family is well known for widespread IC production [64], [65], and several studies have used *Enterobacter* strains to assay the indole-3-acetic acid production pathways [37], [40]. Still, the only report suggesting that enterobacteria produce more ICs than do other taxa of soil bacteria was from our group (Moreira, personal communication). Although there are many reports demonstrating the efficient endophytic colonization of strains belonging to the *Enterobacter* genus, it was never before reported that this genus might be found more often inside the plant tissues than in the rhizosphere in average or rich soils but not in poor soils. As enterobacteria follow an r-strategy for rapid growth and the quick use of resources [64], the low occurrence of these bacteria in poor soils with less resources is understandable. Additionally, although *Enterobacter* strains are known for displaying P solubilization ability [54], [55], it was not reported that they might solubilize less phosphate than do several other soil bacterial genera (Fig. 7).

It is interesting to notice the differences between the *Burkholderia* and *Enterobacter* genera (Fig. 9). Their PGP traits are almost opposed to each other but both are associated with high siderophores production. Their favored environment and niche are directly opposed as well. It seems that these genera follow distinct strategies for survival and plant interaction, and both are successful. A comparative genomic analysis of these genera could return interesting results for soil bacteria life strategies.

Information concerning the *Grimontella* genus is scarce. Its occurrence in plants is restricted to a previous study [12] of sunflower. This genus stands out among the *Enterobacteriaceae* cluster in Fig. 2 because none of the 10 isolates belonging to this genus produced low amounts of ICs (Fig. 9). Similarly to *Enterobacter*, strains from the *Grimontella* genus are good siderophores producers that live endophytically in rich soils (Fig. 7). Further investigation of this genus might reveal it as a very useful biotechnological agent that has, so far, been largely ignored. To find more *Grimontella* strains in the environment, we suggest sampling surface-sterilized sunflower roots from rich soil conditions using an *Enterobacteriaceae*-friendly culture medium. Additionally, deeper investigation of the similarities between *Enterobacter* and *Grimontella* could provide valuable scientific insights. It might be interesting to note, as well, that strains from the *Grimontella* and *Rahnella* genera behaved very similarly, yet only the strains belonging to the *Grimontella* genus were mostly endophytic.

The strains from the *Herbaspirillum* genus presented low scores of PGP traits, and behaved more endophytically in poor soils (Fig. 7). It is possible that this behavior is a response to fertilization: in N-rich soils, plants no longer require bacterial strains to fix nitrogen, and there is a reduced need for endophytic diazotroph colonization [66]. The strains from the *Rhizobium* genus behaved similarly, except that these strains were more frequent in poor soils (both are shown in Fig. 9). This observation has important crop management implications, as it indicates that farmers have nitrogen fixers in their soils but prevent them from being useful due to the addition of N fertilizers. It is interesting to notice that none of *Herbaspirillum* isolates that were analyzed in this work were able to solubilize phosphates (Fig. 7), reinforcing the finding of Estrada *et al*. [61], who first identified a phosphate-solubilizing *Herbaspirillum* strain.

*Pandoreae* strains were previously isolated from contaminated soils [67], [68] and plant rhizospheres [69], [70], and strains from this genus are promising in biodegradation applications [71]. In this study, we found that strains from *Pandoreae,* similarly to strains from *Rhizobium* and *Herbaspirillum* genera, are associated with poor soils, where they showed endophytic behavior (Fig. 7). However, much unlike *Rhizobium* and *Herbaspirillum* strains, these strains were found in the nutrient-solubilizing cluster of Fig. 2, as they were good phosphate solubilizers but were completely unable of producing ICs, one of the most widespread and important PGP traits of soil bacteria. It is possible that the adaptations of these strains to adverse conditions instead of growth hormone production play a key role in their association with plants. Bioprospectors interested in *Pandoreae* biodegradation could consider endophytic bacteria and soil richness conditions in their sampling strategy.

Although bacteria from the *Klebsiella* genus are known for producing ICs [38], fixing nitrogen [72], solubilizing phosphate [55], producing siderophores [56], and actively colonizing the plant rhizosphere [1], there are no reviews regarding their general role in the rhizosphere. Here we illustrate the *Klebsiella* genus as very common in soil and also very adaptable and versatile, with an overall high IC production and a mix of nutrient-solubilizing abilities (Figs. 7 and 9). Strains from this genus were not associated with any environment or colonization niche, although it has already been reported that Klebsiella would be more often found as a rhizospheric than as a endophythic bacteria [73]. *Klebsiella* followed the model for IC production but was the only genus that behaved against the model concerning siderophores production in rich soils and phosphate solubilization in poor soils (Fig. 8). It becomes clear to us that the ecological significance of *Klebsiella* in soils is largely underestimated.

Most of the PGP trait-shifting bacterial genera presented on Fig. 8 followed our model. In richer soils, the ICs production levels increased as the phosphate solubilization and siderophores production abilities decreased, but in poorer soils, the ICs production decreased as the nutrient solubilization increased.

**Figure 10:**
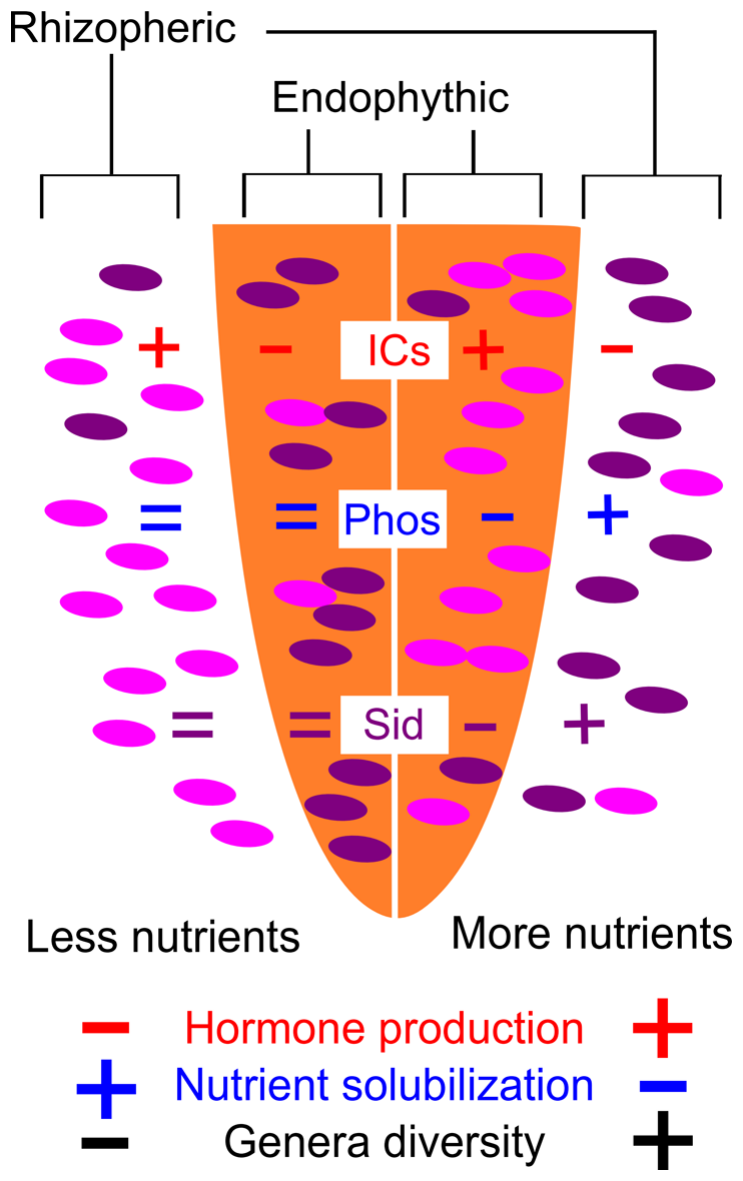
A model to explain the distribution of bacteria displaying different plant growth promotion traits. In soils with fewer nutrients, plants leave the best growth hormone producers in the rhizosphere, while both endophytic and rhizospheric bacteria are good nutrient solubilizers. In soils with more nutrients, the best growth hormone producers are found inside plant roots, but the endophytic bacteria are poor nutrient solubilizers, with the best solubilizers found in the rhizosphere. In addition, genera diversity and growth hormone producers are more abundant in soils with more nutrients, while phosphate solubilizers and siderophores producers are more abundant in soils with fewer nutrients. Siderophores producers and phosphate solubilizers seem to co-occur, while indolic compound producers are clearly opposed to phosphate solubilizers. Plants seem to select bacterial PGP traits according to their nutritional needs: nutrient solubilizers under poor conditions and growth hormone producers under rich conditions.

Based on our data, we updated a previously proposed model that is explained in Fig. 10 and described above. This model helps direct bioprospection for PGPB so that the bacteria (or genes) displaying a trait of interest can be more easily found in the soil and root samples, considering the soil richness and niche occupation by these bacteria. We also found several interesting PGPB-niche-environment interactions at the genus level that could aid PGPB bioprospection by using appropriate selective medium or molecular markers or by directing research questions.

## Conclusions

We propose a model for the occurrence of some plant growth-promoting traits in plant-associated bacteria. This model praises that plants will favor their association with endophytic bacteria according to the nutrient status of the soil, permitting an association with nutrient solubilizers under nutrient-poor conditions or selecting growth hormone producers under nutrient-rich conditions. We also suggest several associations at the genus level, demonstrating where some genera are more likely to be located and which phenotypic traits they should be displaying. This model could be used for directed PGPB bioprospection, so that target PGP traits or bacterial genera can be screened in the right niche and under the right conditions, which is important both for cultivation-dependent and -independent methods, as both are time-consuming and expensive and, therefore, should not blindly sample plants and roots.

## Supporting Information

S1 Fig **Soil chemical characteristics according to PCA cluster classification.** (a) Log-transformed values (average ±1 SE) of Potassium (K), Clay content, Organic matter, and pH for poor, average and rich soils. (b) Rank values (average ±1 SE) of Phosphate (P) content for poor, average and rich soils. Different letters show significant differences.
(TIF)

S2 Fig **Heat map associations of the TCP solubilization (left) and siderophores production (middle) abilities of bacterial isolates with soil conditions and with each other (right), displayed in percentages (a) and adjusted residuals (b).** The legend and interpretation are similar to those of Fig. 4.
(TIF)

S3 Fig **Heat map associations of the TCP solubilization and siderophores production abilities of endophytic (root) and rhizospheric (soil) isolates under each individual soil condition, displayed in percentages (a) and adjusted residuals (b).** The legend and interpretation are similar to those of Fig. 6.
(TIF)

S4 Fig **Heat map associations of bacterial genera and PGP traits (left), soil richness (middle), and occurrence of putative endophytic (Root) and rhizospheric (Soil) bacteria under each soil richness condition (right), displayed in percentages (a) and adjusted residuals (b).** The legend and interpretation are similar to those of Fig. 7.
(TIF)

S5 Fig **PGP traits of some bacterial strains shifted due to the soil richness.** Only those bacterial genera that significantly changed their PGP traits are shown. Each box is a separate chi-square test, displayed in percentages (a) and adjusted residuals (b). The legend and interpretation are similar to those of Fig. 8.
(TIF)

S6 Fig **Heat map associations of the TCP solubilization and siderophores production abilities of endophytic (root) and rhizospheric (soil) isolates of the** ***Burkholderia*** **genus under each individual soil condition (the legend and interpretation are similar to those of** **Fig. 6** **).** Only the *Burkholderia* isolates are displayed here.
(TIF)

S1 Table **Full information of each isolate used in this study.** Includes quantification of plant growth promoting traits, colonization niche, bacterial genera, soil richness, isolate geographical origin, code on PCA plot, and associated plants.
(XLSX)

S2 Table **Chemical characteristics of all soils analyzed in this study.**
(XLSX)

S3 Table **Details of all statistical tests used in this study.** Includes p values, sample sizes, false discovery rate, degrees of freedom, names of the tests, and the figures where they are shown in the paper.
(XLSX)

S1 Text **Additional information on statistical methodology, showing how the tests used in the paper were calculated and interpreted.**
(DOCX)
