# Supplementary figures and images for: A Model to Explain Plant Growth Promotion Traits: A Multivariate Analysis of 2,211 Bacterial Isolates
Source: PLoS One. 2014 Dec 26;9(12):e116020. doi: 10.1371/journal.pone.0116020 (PMC4277451; doi:10.1371/journal.pone.0116020)

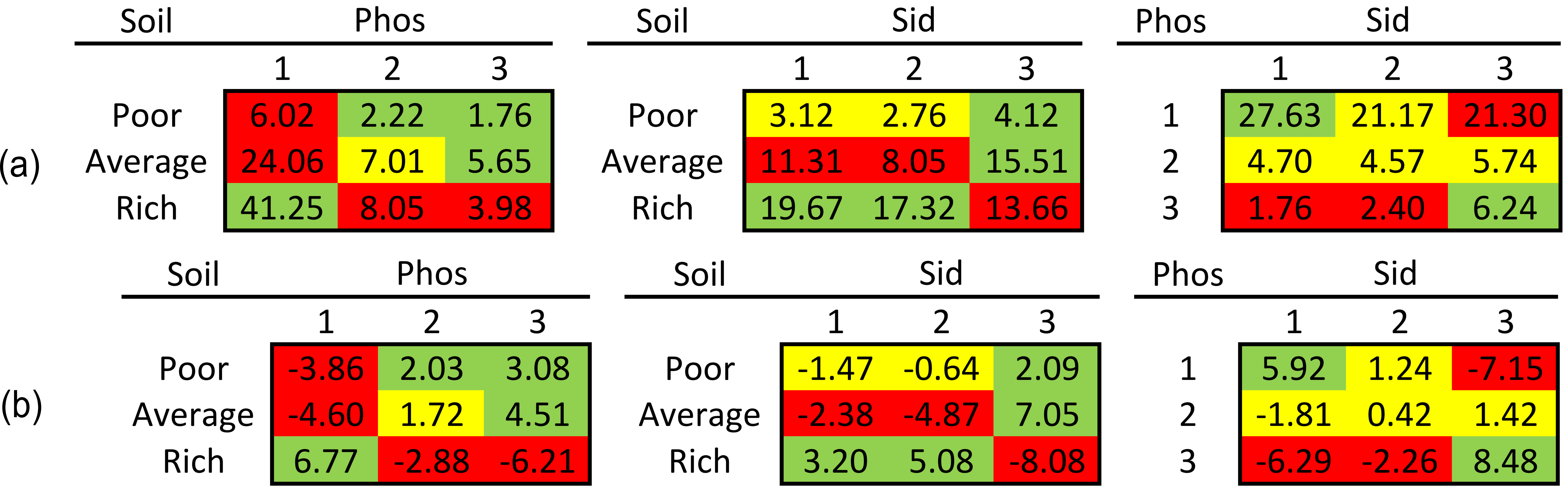

Supplement: S2 Fig — Heat map associations of the TCP solubilization (left) and siderophores production (middle) abilities of bacterial isolates with soil conditions and with each other (right), displayed in percentages (a) and adjusted residuals (b). The legend and interpretation are similar to those of Fig. 4. (TIF) [file pone.0116020.s002.tif]

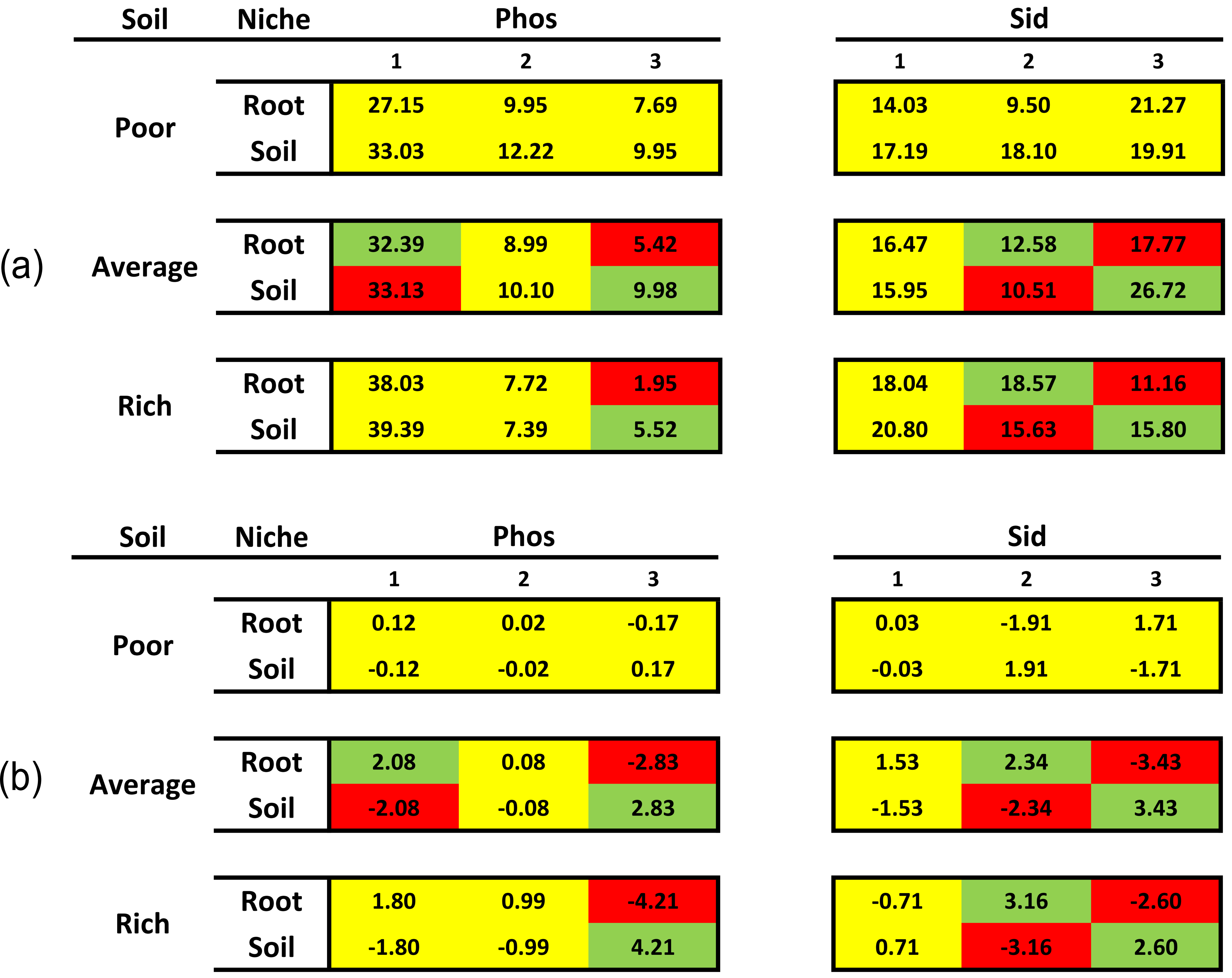

Supplement: S3 Fig — Heat map associations of the TCP solubilization and siderophores production abilities of endophytic (root) and rhizospheric (soil) isolates under each individual soil condition, displayed in percentages (a) and adjusted residuals (b). The legend and interpretation are similar to those of Fig. 6. (TIF) [file pone.0116020.s003.tif]

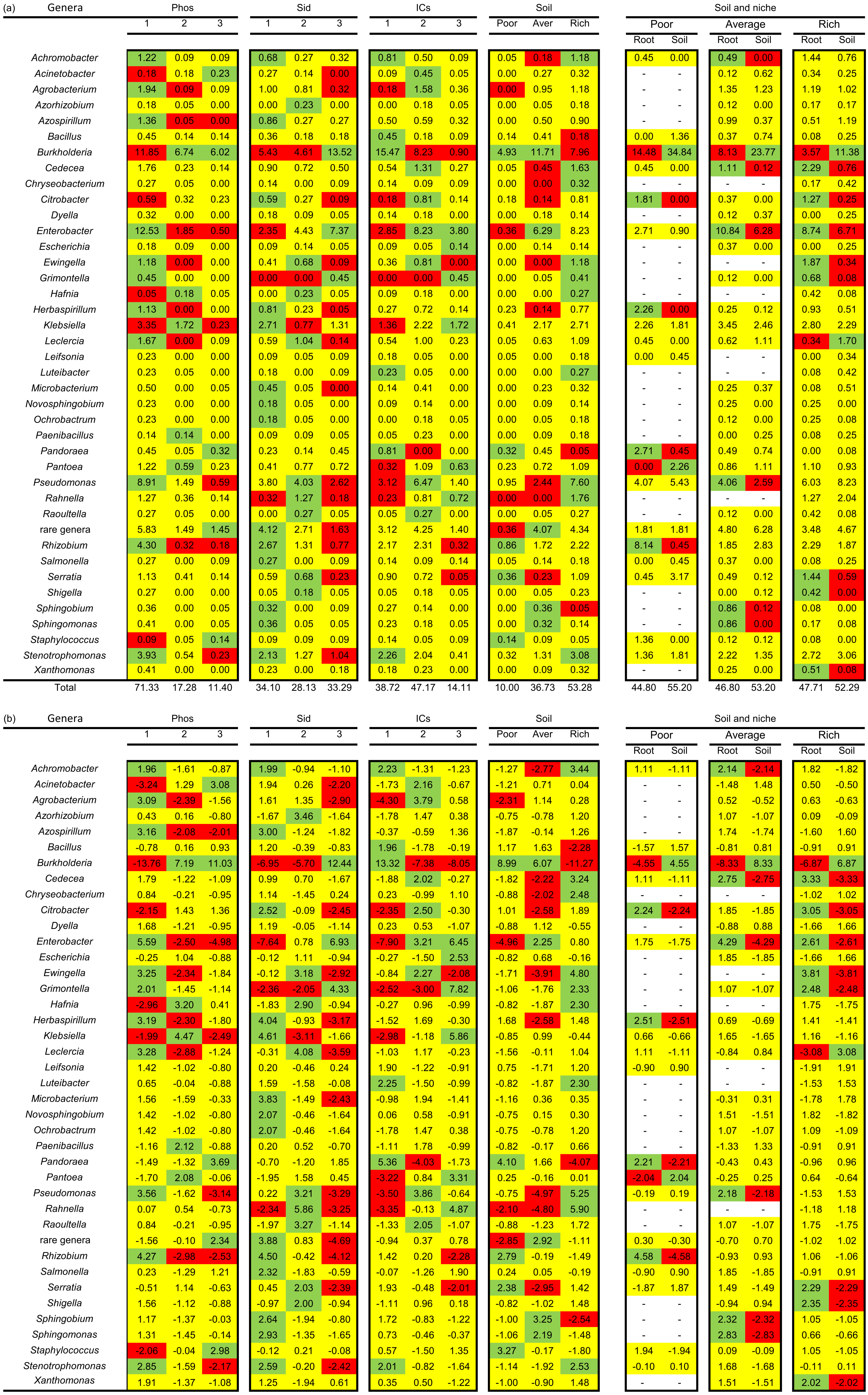

Supplement: S4 Fig — Heat map associations of bacterial genera and PGP traits (left), soil richness (middle), and occurrence of putative endophytic (Root) and rhizospheric (Soil) bacteria under each soil richness condition (right), displayed in percentages (a) and adjusted residuals (b). The legend and interpretation are similar to those of Fig. 7. (TIF) [file pone.0116020.s004.tif]

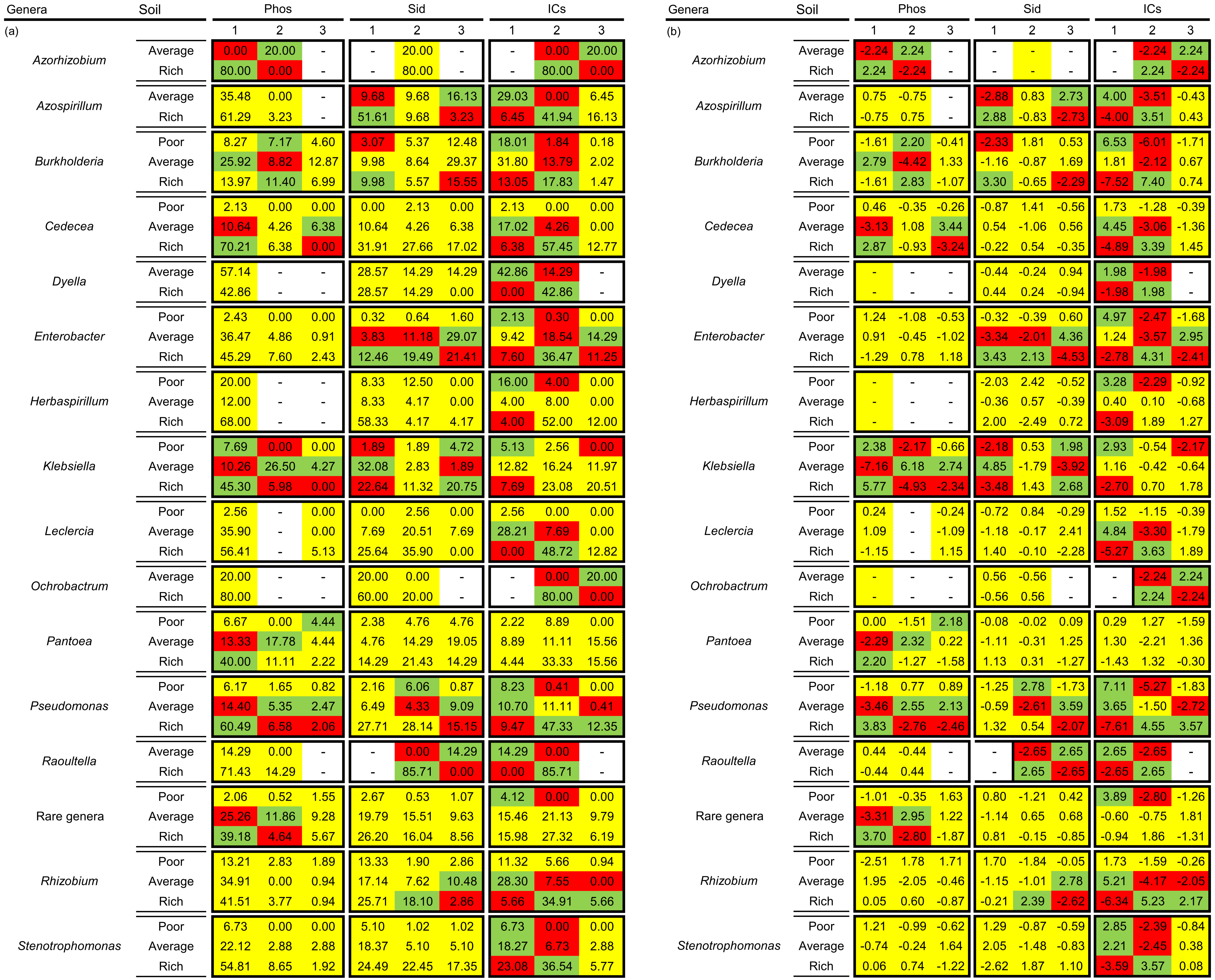

Supplement: S5 Fig — PGP traits of some bacterial strains shifted due to the soil richness. Only those bacterial genera that significantly changed their PGP traits are shown. Each box is a separate chi-square test, displayed in percentages (a) and adjusted residuals (b). The legend and interpretation are similar to those of Fig. 8. (TIF) [file pone.0116020.s005.tif]

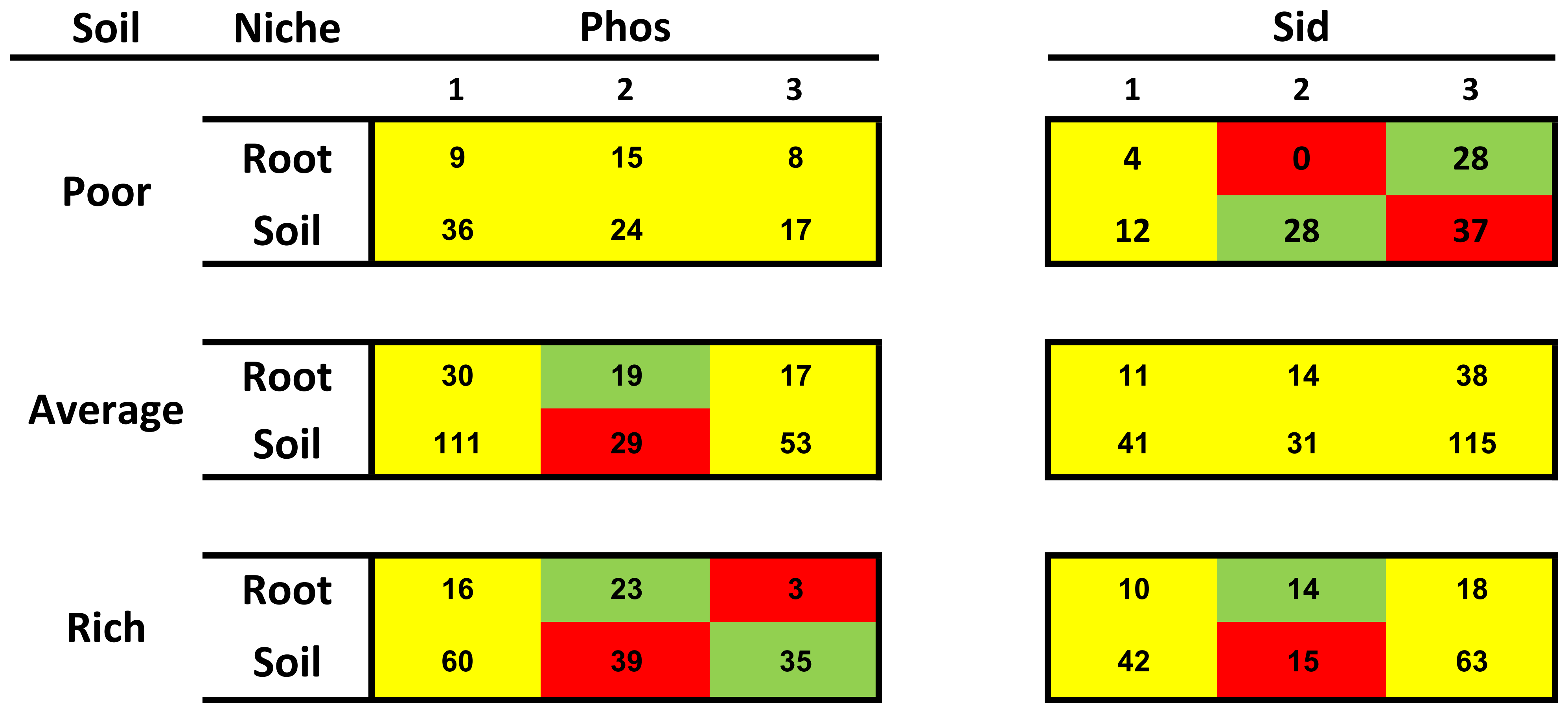

Supplement: S6 Fig — Heat map associations of the TCP solubilization and siderophores production abilities of endophytic (root) and rhizospheric (soil) isolates of the Burkholderia genus under each individual soil condition (the legend and interpretation are similar to those of Fig. 6 ). Only the Burkholderia isolates are displayed here. (TIF) [file pone.0116020.s006.tif]
