# Supplementary material for: A Model to Explain Plant Growth Promotion Traits: A Multivariate Analysis of 2,211 Bacterial Isolates
Source: PLoS One. 2014 Dec 26;9(12):e116020. doi: 10.1371/journal.pone.0116020 (PMC4277451; doi:10.1371/journal.pone.0116020)
Supplement: S1 Text — Additional information on statistical methodology, showing how the tests used in the paper were calculated and interpreted. (DOCX) [file pone.0116020.s010.docx]

**Additional information on statistical methodology, showing how the tests used in the paper were calculated and interpreted.**

***How PCA works***

Principal component analysis is a multivariate dimension reduction method that allows us to reduce complexity of several dimensions (variables) into a few independent dimensions (the principal components, separate axis in the chart). Linear correlations between the variables are calculate to obtain eigenvalues and eigenvectors in a matrix, that once plotted allow easy visualization of a portion of the dataset variation. Subjects in the dataset (different soils in this paper) can then be clustered in different groups, and the continual variables (soil chemical parameters in the paper) reveal their correlation to each other (and to the subjects) according proximity of the lines they represent in the plot. Each principal component explains a defined and additive amount of variation (46% and 23% for principal components 1 and 2, in this paper), with additional principal components (one for each variable) explaining each time less variance, for a total of 100% variance explained. Positive and negative values of the principal components are helpful to separate clusters.

***How CatPCA Works***

Our CatPCA analysis simultaneously shows us several correlations between PGP traits, soil richness, and bacterial genera. Each colored line in the plot indicates an increasing vector, calculated by the values of each variable in accordance to the other variables. Indolic compounds production is considered a continuous numeric variable, while phosphate solubilization, siderophores production and soil richness scores are considered ordinal variables (there is an increasing order in the magnitude of the variables, such as non solubilizers =1, average solubilizers =2 and good solubilizers =3). The different genera are considered as multiple nominal variables, since there is no inherent order between the genera, and thus they do not generate an increasing vector on the plot. Classifying these variables is very important for correct CatPCA construction. The Cronbach alpha value, a reliability measure that represents the scale consistency on two-dimensional plots, was satisfactory (0.769>0.7). To analyze the plot from Figure 2, one can cluster data points from different variables and consider them to be associated or not. It is important to consider direction and inclination of the different vectors between each other, since this could also indicate associations: vectors close to each other are more highly correlated, showing positive correlations if they point to the same side of the plot, but showing negative correlation when pointing to opposite directions. CatPCA differs from PCA as it is able to mix continual and categorical data, but unable to sum the explanation percentage of each dimension due to the presence of multiple nominal variables. PCA assumes linear relationships between numeric variables, but the optimal-scaling approach of CatPCA allows variables to be scaled at different levels. As a result, nonlinear relationships between variables can be modeled. CatPCA, just like PCA, is an unsupervised learning method that does not test hypothesis and does not return p values for associations or correlations. For that end, we used nonparametric statistics, which are supervised learning methods able to test for hypothesis directly.

***How Residual analysis works***

The association heatmaps are composed of independent chi-square tests on the same dataset. Expected number of cases depends on marginal total values – that is, the number of expected isolates at any table cell depends on the number of isolates on that line, column and total isolates on that test [(column total x line total)/sample size]. The difference between observed and expected values return a standardized residual adjusted (not shown) that follows the Z distribution. At values <|1.96|, <|2.58|, <|3.30| and <|6.0|, the residuals indicate significant individual associations with p=0.05, 0.01, 0.001, and 0.000000001 respectively. Our heatmaps show green and red colors for values <|1.96|, but several of the calculated residues had values <|3.30| or <|6.0|. The actual residual values used for the heatmaps are presented on supplementary figures 1 to 4.

When certain genera were not found in a certain condition (several cases on poor soils) we could not calculate their expected occurrence and their associated p values, and thus their corresponding spaces in the heatmap were left empty. Signaling these situations as yellow (which meant equal colonization of rhizosphere and plant tissues) would be misleading, as we had no information of how these genera colonize plants in such environments.

Monte Carlo simulation was used when calculating associations between the different genera and the other PGP traits, niches or environments. This simulation method is used when there’s not enough computational power to determine the exact p value, so it estimates a p value window based on a resampling simulation.

***How Kurskal-Wallis and Mann-Whitney tests work***

As a nonparametric variance analysis test, Kruskal-Wallis and Mann-Whitney operates on variables ranks instead of the variables themselves. The lowest values on a dataset is given rank “1”, the second lowest rank “2” and so forth until the highest value in the dataset (with corrections to ties). Average ranks between treatments are then compared much like in ANOVA or T-test, but with mathematical operations based on sums of ranks. Nonparametric alternatives of parametric tests are usually employed when statistical assumptions are violated (like normal distribution and homogeneity of variances), despite data transformations like Log or square root. This comes at a cost, however, as nonparametric tests have less power (require larger differences between treatments to reject the null hypothesis when it is false) than their parametric versions.

Non-ICs producers were not included in the analysis for two reasons: 1) we were more interested in seeing the actual average production of the isolates in each condition, and inclusion of non-producers would artificially reduce the average of any condition that had more non-producers. 2) If several non-producers were included in the non-parametric rank-sum tests, they would produce several ties of the same value, that could add an unnecessary background noise on the comparisons, lowering the tests efficiency.

***Alternatives approaches on our CatPCA analysis***

Niche occupation by the bacteria was not included in the CatPCA analysis. This was due to three reasons: 1) there is a significant interaction effect between niche and soil richness for all PGP traits, as shown below and proposed by the model. This means that PGP traits of each niche for each PGP trait depends on the environment, and the CatPCA could only show the total average rhizospheric or endophythic isolate characteristics, what prevents us from seeing the interaction. 2) Addition of the niche variable in the CatPCA as it is would only show two points very close to each other and to the center of the plot, explaining very little variance while reducing the reliability of the test due to the addition of another variable. 3) If we compute both the niche and soil richness condition as a single variable (showing 6 points in the plot), what would allow us to see the interaction effect, we would lose the ordinal nature of the soil richness condition (increase from poor to rich) to consider it as a multiple nominal variable (since rhizospheric isolates are no higher or lower than endophytic isolates). This reduces our variance explanation and some of the linearity of our model.

In a reiteration of our CatPCA we also performed analysis of the third dimension. The Cronbach’s alpha value increases to 0.838, we can see that phosphate solubilization slightly leans toward positive values of the Z axis, and the different genera are more distributed, allowing them to be better characterized. But since CatPCA on SPSS software only provides multiple 2D figures, visualization becomes difficult. As our final conclusions were not changed due to this alternative visualization, we did not include it in this work, despite its potential to better visualize some of the genera’s PGP traits.

To better visualize the PGP trait shift of important bacterial genera from the dataset, we created additional CatPCA plots (Fig. S1). There, a single genus is visually displayed, showing all isolates haplotypes from that genus. All the other 39 genera were visually suppressed, but still take part in mathematical construction of the plot. It can be seen that strains belonging to *Pseudomonas* genus span across all environments and PGP vector levels, while those belonging to *Grimontella* genus were located well above, always due to their high ICs production levels – 82 to 220 μg of ICs ml^-1^

***False Discovery Rate***

When several tests are performed in any single paper, there is a cumulative chance of erroneously rejecting the null hypothesis (type I error). This happens because each test is adding a cumulative 5% chance that the differences on the observed data might be occurring by chance (for p<0.05). The chance of erroneously rejecting the null hypothesis is defined by the equation 1-(1-α)*^M^*, where α = significance level chosen for the tests and *M* is the total number of tests in the paper. In this work, 120 hypothesis tests were performed, and the chance of erroneously rejecting the null hypothesis at least one time is 99.78%. To correct the global type I error, we used a False Discovery Rate of 10%. This procedure limits the wrongly rejected null hypothesis to 10% of the significant data, and has more power compared to Bonferroni Correction, that is too conservative for our dataset. Using this tool, we considered as non-significant one result of p=0.042 when analyzing the bacterial genera PGP traits shifts. The complete dataset (and all P values for all 120 tests) are represented on Table S3. FDR was calculated in SPSS software with the following syntax:

“sort cases by pvalue (a).

compute i=$casenum.

sort cases by i (d).

compute q=.10.

compute m=max(i,lag(m)).

compute crit=q*i/m.

compute test=(pvalue le crit).

compute test=max(test,lag(test)).

execute.

formats i m test(f8.0) q (f8.2) crit(f8.6).

value labels test 1 'Significant', 0 'Not Significant'.”
